# Factors Associated with Self-Reported Changes in Alcohol Use among Young Adults during the COVID-19 Pandemic: A Comparative Analysis between Canada and France

**DOI:** 10.3390/ijerph192416694

**Published:** 2022-12-12

**Authors:** Pierre-Julien Coulaud, Guillaume Airagnes, Kai McGrath, Naseeb Bolduc, Karine Bertrand, Marie Jauffret-Roustide, Rod Knight

**Affiliations:** 1 Department of Medicine, University of British Columbia, Vancouver, BC V6T 1Z4, Canada; 2 British Columbia Centre on Substance Use, Vancouver, BC V6Z 2A9, Canada; 3 AP-HP. Centre-Université Paris Cité, DMU Psychiatrie et Addictologie, 75006 Paris, France; 4 INSERM, Population-Based Epidemiological Cohorts, 94800 Villejuif, France; 5 Faculty of Medicine and Health Sciences, Université de Sherbrooke, Longueuil, QC J1K 2R1, Canada; 6 Centre d’Étude des Mouvements Sociaux (EHESS/CNRS UMR8044/INSERM U1276), 75244 Paris, France; 7 Baldy Center on Law and Social Policy, Buffalo University, Buffalo, NY 14260, USA; 8 Centre for Gender and Sexual Health Equity, Vancouver, BC V6Z 2K5, Canada; 9 École de Santé Publique de l’Université de Montréal, Montreal, QC H3N 1X9, Canada

**Keywords:** alcohol, young adults, COVID-19, depressive symptoms, Canada, France, online survey

## Abstract

While the COVID-19 pandemic impacted young adults’ alcohol use patterns, little is known about how changes in alcohol use may differ across different settings. Our objective was to identify and compare factors associated with changes in alcohol use among young adults in Canada and France during the first year of the COVID-19 pandemic. We conducted an online cross-sectional survey in October–December 2020 with young adults aged 18–29 (*n* = 5185) in Canada and France. In each country, weighted multinomial logistic regressions were performed to identify factors associated with self-reported decrease and increase in alcohol use separately (reference: no change). Respectively, 33.4% and 21.4% reported an increase in alcohol use in Canada and France, while 22.9% and 33.5% reported a decrease. Being 25–29 was a predictor of decrease in Canada, while living away from family was associated with an increase in France. In both countries, participants were more likely to report an increase if they reported depressive symptoms, smoking tobacco, or cannabis use. Conversely, those who had been tested for COVID-19 and those who were highly compliant with COVID-19 preventive measures were more likely to report a decrease. Efforts are needed to develop alcohol use interventions for young adults, including in ways that prioritize those with mental health challenges.

## 1. Introduction

A growing body of evidence indicates that the COVID-19 pandemic has impacted young adults’ alcohol use patterns and behaviors [1,2]. A recent review examining changes in youth substance use documented that most studies comparing data before and during the pandemic observed a decrease in quantity and frequency of alcohol use, as well as lower prevalence of alcohol use disorders [1]. Most of these studies explained this decline as a consequence of the implementation of COVID-19 public health measures (e.g., closure of bars and universities, limiting social gatherings), which reduced the opportunities for young adults to socialize while consuming alcohol [3,4,5]. Conversely, other studies among young adults and students reported an increase in alcohol use [6,7,8] with a wide range of associated factors, including feelings of boredom, having more time, and as a coping strategy for mental health issues (e.g., depression, isolation) [7,9,10]. These findings suggest a more nuanced picture regarding the potential for bidirectional effects of the pandemic on young adult alcohol patterns [11] and/or substantial variations across study settings [2,12,13,14], thus underscoring the need for research that investigates changes in alcohol use in both directions and across different settings.

In the existing COVID-19 literature among young adults, three main sets of factors have been reported to be associated with diverse changes in alcohol use. First, socio-demographic factors have been identified, such as age [6,15,16], gender [6,15,17,18], ethno-racial identity [15,19,20], and living situation [6,15,21,22,23]. For example, students who moved to live with their parents were less likely to drink alcohol compared to those who continued living in their pre-COVID residences [23]. A second set of factors feature the COVID-19 pandemic and associated public health measures. Previous studies identified how COVID infection, episodes of quarantine, in-person social interactions, and lower levels of adherence to COVID-19 measures were all associated with increased alcohol use [15,17,18,24,25,26]. Other studies also suggest that young adults who have been affected by the negative economic consequences of the pandemic (e.g., employment loss) had a greater likelihood of increased alcohol use [21,27]. A third set of factors were related to mental health and prior substance use. Previous studies showed that young adults who have experienced mental health issues (e.g., depression, anxiety, loneliness), those with pre-pandemic high drinking levels, and those using other substances (e.g., smoking cigarettes) had a greater risk of reporting an increase in alcohol use [27,28,29].

Despite emerging research documenting variations in changes of alcohol use across study settings [2,12,13,14], it remains unclear as to how and why these previously identified factors occur within and across countries. Most international research works in this area have pooled their entire sample when reporting findings [30,31], thereby limiting understandings about how contextual factors (e.g., socio-cultural habits, alcohol regulatory frameworks) may impact alcohol use patterns among young adult populations during the COVID-19 pandemic. For example, alcohol use was described as a social inclusion ritual in France, deeply embedded in the “French culture” (frequently associated with the notion of “art de vivre”) [32], while in Canada, limited alcohol control policies have been implemented, including limited promotion of health and safety messaging about alcohol [33]. Further international comparative analyses are therefore needed to develop context-sensitive and population-specific alcohol interventions for young adults. 

In order to investigate the contextual factors influencing alcohol use patterns among young adults, we used data collected from the *France Canada Observatory on COVID-19, Youth health and Social well-being (FOCUS)* study (www.focus-study.me, 2 December 2022), a cross-sectional online survey that aims to investigate the effect of the COVID-19 pandemic on social and health outcomes among young adults living in two high-income countries with socio-cultural and regulatory differences regarding alcohol: Canada and France. The objective of the current study is therefore to identify and compare the factors associated with changes in alcohol use among young adults living in Canada and France during the first year of the COVID-19 pandemic.

## 2. Materials and Methods

### 2.1. Study Design and Context

The FOCUS online survey was conducted among young adults aged 18 to 29 years and living in Canada or France during the second wave of the pandemic, i.e., from 8 October to 23 December 2020.

Canada and France have distinct histories, cultural habits, and policies regarding alcohol consumption. France has one of the highest consumption rates in the world, with 12.3 L of pure alcohol consumed per capita per year compared to 8.9 L in Canada. In addition, 31% of the French population aged 15 and over engage in binge drinking at least once a month compared to 21% in Canada [34]. Alcohol in France is strictly regulated by the Évin law passed in 1991, but in practice, the power of French alcohol lobbies constitutes a limitation for implementing efficient prevention policies [35]. While alcohol is available in any supermarket or convenience store for people over 18 and can be consumed in public spaces in France (except when local laws are introduced to ban public drinking in certain areas/cities), alcohol can only be purchased at licensed liquor stores in Canada, and public drinking is prohibited in all provinces (except for designated parks/spaces). In Canada, the minimum legal drinking age varies depending on the province or territory of residence: 18 years in Alberta, Manitoba, and Québec, and 19 years in the rest of Canada. Policies regarding alcohol advertising also vary between Canada and France, with stricter regulations on sales promotion and more health warnings on advertisements in France compared to Canada [36,37].

During the pandemic, the implementation of public health preventive measures to limit the spread of COVID-19 (e.g., lockdown, curfew) has led to alcohol policy changes in Canada and France [38]. In both countries, alcohol retailers were considered as essential services and remained open during lockdown periods. While closures of restaurants/bars and restrictions of social gatherings have limited sales, other policy changes have been implemented in many countries, including Canada and France, to allow online sales, takeout purchases, and home delivery of alcohol [38]. In Canada, there were some provincial differences in alcohol policy changes. For example, Ontario and British Columbia have reduced minimum unit pricing for licensed establishments and increased outdoor spaces for alcohol consumption, while the territories of Canada did not permit alcohol sales through online services, takeout, or home delivery [39].

### 2.2. Data Collection and Participants

The present analysis used part of the data collected in the FOCUS study. The FOCUS online questionnaire was designed to collect data on socio-demographics, COVID-19 perceptions and experiences, access to healthcare services, and health outcomes, including mental health and substance use (e.g., alcohol use). The participants of the FOCUS study were recruited using a convenience sampling method, primarily through online posts and advertisements on social media (e.g., Facebook, Instagram), and on websites of university partners, press articles, and word of mouth. The eligibility criteria included: (1) being of legal age in the jurisdiction of residence at the time of the survey (i.e., 18 or 19 years old depending on the Canadian province or territory, and 18 years old in France) and no more than 29 years; (2) residing in Canada or France; and (3) being able to complete the survey in English (Canada) or French (either country). Survey data were collected using *Qualtrics*. Informed consent was requested on the introductory web page and was required prior to accessing the survey questionnaire. The study was approved by University of British Columbia’s Behavioral Research Ethics Board (H20-02053).

### 2.3. Measures

#### 2.3.1. Outcome

Our study outcome, self-reported changes in the frequency of alcohol use, was assessed by asking the following question: “In the last six months, has the frequency of your alcohol use changed because of COVID-19?”. Participants were provided with three response options: “yes, it has decreased/is less because of COVID-19”, “no, it has not changed (or changed for reasons other than COVID-19)”, and “yes, it has increased/is more because of COVID-19”.

#### 2.3.2. Exposure Variables

Three groups of exposure variables were considered.

*Socio-demographic characteristics* included age, gender identity, sexual orientation, province/region of residence, area of living, foreign-born status (born in Canada or in Metropolitan France), education, employment status, and living arrangement. In Canada, ethno-racial identity was collected using the Canadian Institute for Health Information standards and dichotomized into three categories: non-racialized, Indigenous, and racialized non-Indigenous (see notes of Table 1 for further details). Due to the prohibition of collecting such data in France, we created the variable “descendants of immigrants” based on the definition from the French National Institute of Statistics and Economic Studies [40] as a proxy for ethno-racial identity. French participants who reported that at least one of their parents or two of their grandparents from the same side were born outside France or Europe were considered as descendants of immigrants.

*COVID-19-related stressors*. Participants were asked if they had been tested for COVID-19 in the past six months. Due to the limited sample size of participants who reported a positive test result, we were not able to investigate the association with COVID-19 status in the present analysis. We also asked participants if they took any preventive measures to decrease their risk of contracting or transmitting COVID-19 in the past six months (each measure checked was scored as 1). To assess their level of compliance with COVID-19 preventive measures, we created a score (ranging from 0 to 5) based on the following five socially restrictive measures: staying home for work or school, only taking essential trips, avoiding social gatherings of over 10 people, avoiding meeting friends, and maintaining a social bubble at home. A total score was obtained by summing all these five measures. Interquartile intervals were used to categorize participants in three groups: low (score 0–2), medium (score 3–4), and high level of compliance (score of 5). Lastly, we asked participants if they have lost any income because of measures taken as part of the COVID-19 pandemic.

*Mental health and substance use factors*. Depressive symptoms within the past two weeks were measured using the nine-item Patient Health Questionnaire (PHQ-9) [41]. A total score ranging from 0 to 27 was obtained by summing all items and interpreted as follows: minimal (0–4), mild (5–9), moderate (10–14), moderately severe (15–19), and severe (20–27). Harmful use of alcohol (score > 8) was measured using the Alcohol Use Disorders Identification Test (AUDIT) [42]. The frequency of smoking cigarettes in the last six months was also recorded and dichotomized in three categories: regular users (“every day”, “multiple times per day”), occasional users (“once or twice per week”, “every month”, or “less than monthly”), non-users (“never”). Participants were asked if they have used cannabis in the last six months.

### 2.4. Statistical Analysis

To improve the representativeness of our sample, we applied survey weights in all the analyses to adjust the sample distribution to the respective young adult population in each country by age, gender, and province/region of residence (see Ref [43] for more details). To account for this survey design, weighted Chi-square tests using Rao–Scott second-order correction were performed to identify differences in the response distribution between groups of changes in alcohol use (i.e., no change, decrease, and increase) [44].

Multinomial logistic regression models were used to identify the factors associated with a decrease or increase in alcohol use. In both sets of analyses, reporting no change was considered as the reference group. All the analyses were stratified for the country of residence (i.e., Canada or France). All exposure variables were entered in multivariable models without a variable selection procedure, except for the AUDIT score, which was excluded because it was overly correlated with our study outcome, as demonstrated in previous COVID-19 studies [31,45]. All the analyses were performed using R version 4.0.3.

## 3. Results

### 3.1. Study Population

Of the total 8424 young adults who participated in the FOCUS survey, 744 (8.8%) were excluded from the present analysis because of missing data on age, gender, or province/region of residence. Participants who did not complete the questions about alcohol use (20.2%) and those who reported being non-alcohol users (i.e., non-use in the last 6 months) (12.2%) were also excluded. Thus, a total of 5185 young adults were included in the present study, with 2793 (53.8%) living in Canada and 2392 in France (46.2%). Study participants’ characteristics are described in Table 1.

**Table 1:** Characteristics of the study population after weighting (overall and by groups of changes in alcohol use) in Canada (*n* = 2793) and France (*n* = 2392).

		Canada					France				
		Total, *n* (Column %)	Self-Reported Change in Alcohol Use, Row %				Total, *n* (Column %)	Self-Reported Change in Alcohol Use, Row %			
			No Change	Decrease	Increase	*p*-Value		No Change	Decrease	Increase	*p*-Value
**All participants**		2793 (100)	43.6	22.9	33.4		2392 (100)	45.1	33.5	21.4	
***Socio-demographic characteristics***											
**Age (years)**						0.006					<0.001
	18–19	428 (15.3)	47.0	24.1	29.2		409 (17.1)	48.7	34.7	16.9	
	20–24	1190 (42.6)	43.9	24.2	31.9		982 (41.1)	42.3	37.6	20.2	
	25–29	1175 (42.1)	42.2	21.3	36.6		1000 (41.8)	46.4	28.9	24.7	
**Gender identity**						<0.001					0.004
	Woman	1237 (44.3)	46.9	24.3	28.9		1110 (46.4)	47.3	33.6	19.1	
	Man	1333 (47.7)	40.6	21.2	38.3		1174 (49.1)	43.0	33.7	23.3	
	Non-binary/other gender identity ^$^	223 (8)	43.5	26.0	30.0		108 (4.5)	44.4	29.6	25.9	
**Sexual orientation**						0.13					0.2
	Straight/heterosexual	1567 (56.1)	45.0	21.6	33.4		1597 (66.8)	45.5	33.4	21.2	
	Bisexual	548 (19.6)	43.2	23.9	32.8		321 (13.4)	40.8	36.1	23.1	
	Other sexual minority ^£^	611 (21.9)	42.4	26.0	31.8		419 (17.5)	47.7	30.8	21.5	
	Missing data	67 (2.4)	26.9	17.9	55.2		55 (2.3)	36.4	43.6	21.8	
	**Province of residence** **(only Canada)** ***^ ¶^***					<0.001					
	Ontario	1011 (36.2)	45.2	22.7	32.0						
	Atlantic	175 (6.3)	49.1	21.1	29.7						
	British Columbia	384 (13.7)	41.7	25.5	32.8						
	Prairies	567 (20.3)	45.1	18.0	36.9						
	Quebec	633 (22.7)	39.0	26.7	34.3						
	Territories	23 (0.8)	56.5	21.7	21.7						
	**Region of residence** **(only France)** ***^ ^^***										<0.001
	Ile-de-France						589 (24.6)	41.3	38.4	20.4	
	Nord Est						474 (19.8)	42.6	32.5	24.9	
	Ouest						403 (16.8)	49.9	33.3	16.9	
	Sud Est						543 (22.7)	47.3	29.7	23.2	
	Sud Ouest						382 (16)	45.8	33.0	21.2	
**Area of living**						<0.001					<0.001
	Large urban center	1728 (61.9)	41.3	24.0	34.8		1254 (52.4)	42.3	36.8	20.8	
	Medium city or town	543 (19.4)	45.7	23.6	30.8		498 (20.8)	46.6	30.5	22.9	
	Small city or rural area	522 (18.7)	49.4	18.8	31.8		640 (26.8)	49.2	29.2	21.6	
	**Ethno-racial identity (only Canada)** ***^ §^***					<0.001					
	Non-racialized	2391 (85.6)	43.9	22.0	34.0						
	Indigenous	132 (4.7)	45.5	12.9	41.7						
	Racialized, non-Indigenous	239 (8.6)	43.1	37.2	20.1						
	Missing data	31 (1.1)	19.4	22.6	58.1						
	**Descendants of immigrants (only France)**										0.049
	No						2017 (84.3)	44.5	34.0	21.5	
	Yes						296 (12.4)	50.7	30.7	18.9	
	Missing data						79 (3.3)	39.2	30.4	30.4	
**Foreign-born status**						<0.001					0.2
	No	2487 (89)	44.2	21.8	34.0		153 (6.4)	50.3	27.5	21.6	
	Yes	306 (11)	38.9	32.4	28.8		2237 (93.5)	44.7	33.8	21.4	
	Missing data	5 (0.2)	60.0	0.0	40.0		3 (0.1)	0.0	33.3	66.7	
**Education**						<0.001					0.005
	High school or college	1073 (38.4)	47.4	18.2	34.4		808 (33.8)	48.5	31.7	19.7	
	University (some, completed, or above)	1714 (61.4)	41.2	25.9	32.8		1574 (65.8)	43.3	34.4	22.3	
	Missing data	6 (0.2)	50.0	33.3	33.3		10 (0.4)	40.0	30.0	40.0	
**Employment status**						<0.001					<0.001
	Employed	1158 (41.5)	44.3	21.1	34.7		864 (36.1)	47.6	30.3	22.1	
	Student (incl. employed)	1316 (47.1)	41.8	25.5	32.7		1244 (52)	43.5	37.1	19.4	
	Unemployed	308 (11)	50.0	16.9	33.1		278 (11.6)	44.2	27.3	28.1	
	Missing data	10 (0.4)	20.0	70.0	10.0		6 (0.3)	50.0	16.7	50.0	
**Living arrangements**						<0.001					<0.001
	Alone	407 (14.6)	48.2	19.7	32.2		774 (32.4)	42.8	34.4	22.9	
	With family members	944 (33.8)	45.4	23.7	30.9		691 (28.9)	49.1	33.7	17.2	
	With partner/ spouse	728 (26.1)	45.5	18.0	36.5		538 (22.5)	47.4	29.6	23.0	
	With friends/ roommates	714 (25.6)	37.0	28.7	34.3		386 (16.1)	39.1	36.8	24.1	
	Missing data	0 (0)	0.0	0.0	0.0		4 (0.2)	75.0	0.0	25.0	
***COVID-19-related stressors***											
	**Experience of COVID-19 testing**					<0.001					<0.001
	No	1795 (64.3)	46.5	21.5	32.0		1369 (57.2)	48.0	29.9	22.1	
	Yes	990 (35.4)	38.5	25.6	36.0		1013 (42.3)	41.3	38.2	20.4	
	Missing data	7 (0.3)	42.9	14.3	42.9		10 (0.4)	30.0	30.0	40.0	
	**Level of compliance with COVID-19 social restrictions measures**					<0.001					<0.001
	Low	684 (24.5)	51.0	15.6	33.2		1323 (55.3)	47.0	30.2	22.8	
	Medium	1087 (38.9)	42.9	23.9	33.1		741 (31)	43.9	33.6	22.5	
	High	1019 (36.5)	39.5	26.7	34.0		321 (13.4)	39.6	46.1	14.3	
	Missing data	3 (0.1)	33.3	33.3	33.3		8 (0.3)	50.0	50.0	0.0	
	**Income loss due to the COVID-19 pandemic**					<0.001					<0.001
	No	1332 (47.7)	48.2	22.0	29.9		1759 (73.5)	46.5	34.2	19.3	
	Yes	1461 (52.3)	39.5	23.8	36.7		633 (26.5)	41.1	31.3	27.5	
***Mental health and substance*** ***use factors***											
**Depressive symptoms**						<0.001					<0.001
	Mild	713 (25.5)	42.5	25.7	31.8		785 (32.8)	44.2	34.4	21.4	
	Minimal	397 (14.2)	59.7	17.1	23.4		501 (20.9)	55.3	33.7	11.2	
	Moderate	635 (22.7)	42.2	24.4	33.5		521 (21.8)	45.3	31.3	23.4	
	Moderate–severe	474 (17)	40.1	22.4	37.6		306 (12.8)	39.5	32.7	27.8	
	Severe	525 (18.8)	38.3	21.5	40.4		217 (9.1)	32.7	35.0	32.3	
	Missing data	47 (1.7)	42.6	31.9	25.5		61 (2.6)	44.3	36.1	19.7	
**Tobacco use**						<0.001					<0.001
	Non-use	2184 (78.2)	46.7	23.5	29.7		1166 (48.7)	51.4	34.0	14.6	
	Occasional smoking	394 (14.1)	35.3	22.6	42.4		420 (17.6)	38.1	41.7	20.2	
	Regular smoking	203 (7.3)	26.6	18.2	55.2		789 (33)	39.2	28.4	32.4	
	Missing data	11 (0.4)	45.5	9.1	45.5		18 (0.8)	55.6	22.2	16.7	
**Cannabis use**						<0.001					<0.001
	No	1057 (37.8)	51.4	25.4	23.2		1395 (58.3)	50.0	34.3	15.7	
	Yes	1727 (61.8)	39.0	21.5	39.5		984 (41.1)	38.2	32.2	29.6	
	Missing data	9 (0.3)	22.2	11.1	66.7		13 (0.5)	46.2	30.8	23.1	

Notes: Survey weights were used in the calculation of all percentages. The *p*-values are from the weighted Chi-square tests using Rao and Scott’s second-order correction to test the independence of all three groups of changes in alcohol use (i.e., no change, decrease, increase) for participants’ characteristics. ^$^ Other gender identity included intersex, two-spirit (only for Canada), and other gender identity with an open-text box. ^£^ Other sexual minority included asexual, pansexual, queer, two-spirit (only for Canada), and other sexual identity with an open-text box. ^§^ Participants who selected any ethno-racial identity (one or more) other than white or Indigenous were classified as racialized, non-Indigenous. The category “non-racialized” includes young adults who selected “white” only and those who reported “white and Latino” or “white and Middle-Eastern” as per the definition in the Canadian Employment Equity Act. The Indigenous category includes those who self-identify as First Nations, Métis, Inuk/Inuit descents. ^¶^ Atlantic: New Brunswick, Newfoundland and Labrador, Prince Edward Island, Nova Scotia; Prairies: Alberta, Manitoba, Saskatchewan; Territories: Nunavut, Yukon, Northwest Territories. ^^^ Nord Est: Grand-Est, Hauts-de-France, Bourgogne Franche-Comté; Sud Est: Auvergne-Rhône-Alpes, Provence-Alpes-Côte-d’Azur, Corse; Sud Ouest: Nouvelle Aquitaine, Occitanie; Ouest: Bretagne, Centre Val-de-Loire, Pays de la Loire, Normandie. Participants from overseas departments were included in the sub-category Sud Est.

### 3.2. Prevalence of Self-Reported Change in Alcohol Use

**Figure 1:**
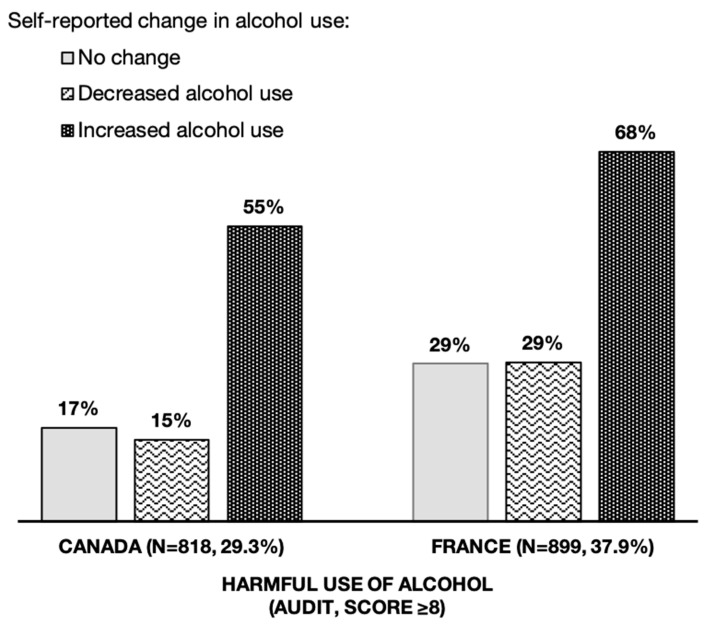
Self-reported change in alcohol use in the last 6 months and harmful use of alcohol among young adults in Canada (*n* = 2793) and France (*n* = 2392).

Overall, most participants (44.3%) reported no change in their alcohol use in the previous 6 months (Table 1). One-third of participants self-reported either a decrease or an increase in their alcohol use (i.e., 27.8% and 27.9%, respectively). A higher proportion of participants reporting an increase was observed in Canada (33.4%) compared to France (21.5%). As shown in Figure 1, respondents with harmful alcohol use were three times more likely in the Canadian sample and two times more likely in the French sample to report an increase in their alcohol use.

As described in Table 1, the highest proportions of increased alcohol use in both countries were found in those smoking tobacco regularly (Canada: 55.2%, France: 32.4%), those reporting cannabis use (Canada: 39.5%, France: 29.6%), and those reporting severe depressive symptoms (Canada: 40.4%, France: 32.3%). In Canada, participants who identified as men and those who identified as Indigenous were the sub-groups who reported the greatest increase in alcohol use (38.3% and 41.7%, respectively). Conversely, racialized young adults (37.2%) and those who were born outside Canada (32.4%) were the most likely to report decreases in alcohol use.

In France, more than 40% of young adults who reported occasionally smoking tobacco (41.7%) and those who had a high level of compliance with COVID-19 social restrictions measures (46.1%) reported a decrease in their alcohol use. In addition, more than half of those who indicated minimal depressive symptoms were more likely to report no change in their alcohol use in both countries (Canada: 59.7%, France: 55.3%). In Canada, the highest rates for no changes in alcohol use were reported among participants living in the territories of Canada (56.5%), while in France, no changes in alcohol use were highest among descendants of immigrants (50.7%) and among those who were born in Metropolitan France (50.3%).

### 3.3. Socio-Demographic Characteristics Associated with Self-Reported Changes in Alcohol Use

The factors associated with changes in alcohol use in the multivariable analyses are reported in Table 2. In both countries, participants who identified as men, compared with women (Canada: adjusted odds ratio (AOR) [95% confidence interval] 1.52 [1.28–1.82], *p* < 0.001; France: AOR 1.45 [1.18–1.78], *p* < 0.001), were more likely to report an increase in their alcohol use. In France, this association was also observed with a decrease for men (AOR: 1.28 [1.08–1.51], *p* = 0.004). Being racialized in Canada (AOR: 1.44 [1.04–1.99], *p* = 0.027) was associated with decreased alcohol use, as well as having a high level of education in both countries (Canada: AOR 1.58 [1.27–1.96], *p* < 0.001; France: AOR 1.47 [1.19–1.82], *p* < 0.001). In the French sample, having a high level of education was also associated with an increase (AOR: 1.48 [1.14–1.91], *p* = 0.003). In Canada, those living in Québec had higher odds of reporting a decrease (AOR: 1.48 [1.15–1.90], *p* = 0.002) and an increase in alcohol use (AOR: 1.41 [1.12–1.79], *p* = 0.004). Participants aged 25–29 had higher odds of reporting an increase in alcohol use compared to the youngest age group (i.e., 18–19) in the Canadian sample (AOR: 1.61 [1.19–2.19], *p* = 0.002). In France, young adults who reported an increase in their alcohol use were more likely to reside in a medium city than in a large urban center (AOR: 1.32 [1.03–1.70], *p* = 0.031) and to live alone (AOR: 1.67 [1.25–2.23], *p* < 0.001) or with a partner (AOR: 1.56 [1.16–2.10], *p* = 0.004) or with roommates (AOR: 2.00 [1.45–2.75], *p* = 0.031) compared to those living with parents.

### 3.4. COVID-19-Related Stressors Associated with Self-Reported Changes in Alcohol Use

In both countries, participants who had been tested for COVID-19 (Canada: AOR 1.33 [1.10–1.59], *p* = 0.002; France: AOR 1.36 [1.16–1.59], *p* < 0.001) and those who reported a high (Canada: AOR 2.15 [1.64–2.82], *p* < 0.001; France: AOR 2.17 [1.71–2.76], *p* < 0.001) or a medium level of compliance with COVID-19 preventive measures had higher odds of reporting a decrease in alcohol use. In Canada, associations with both an increase and a decrease were found for being tested for COVID-19 (increase: AOR 1.2 [1.01–1.43], *p* = 0.036; decrease: AOR 1.33 [1.10–1.59], *p* = 0.002) and those reporting a high level of COVID-19 compliance (increase: AOR 1.39 [1.10–1.75], *p* = 0.006; decrease: AOR 2.15 [1.64–2.82], *p* < 0.001). Lastly, income loss was associated with both decreased (AOR: 1.34 [1.12–1.60], *p* < 0.001) and increased alcohol use (AOR: 1.42 [1.21–1.68], *p* < 0.001) in Canada.

### 3.5. Mental Health and Substance Use Factors Associated with Self-Reported Changes in Alcohol Use

Mental health and substance use factors were all significantly associated with an increase in alcohol use in both countries, including reporting severe depressive symptoms (Canada: AOR 1.52 [1.17–1.97], *p* = 0.001; France: AOR 1.96 [1.41–2.73], *p* < 0.001), smoking tobacco occasionally (Canada: AOR 1.43 [1.12–1.82], *p* = 0.004; France: AOR 1.52 [1.13–2.04], *p* = 0.005), to a greater extent for regular tobacco users (Canada: AOR 2.82 [1.96–4.05], *p* < 0.001; France: AOR 2.13 [1.65–2.76], *p* < 0.001), and reporting cannabis use (Canada: AOR 2.00 [1.67–2.38], *p* < 0.001; France: AOR 1.83 [1.45–2.32], *p* < 0.001). In France, a significant association with increased alcohol use was also found in those reporting moderate-to-severe depressive symptoms (AOR 1.37 [1.02–1.85], *p* = 0.038).

## 4. Discussion

Overall, more than half of the young adults who participated in the FOCUS survey reported a decrease or an increase in their alcohol use during the first year of the COVID-19 pandemic. These proportions differed slightly between the Canadian and French samples, with a higher proportion of participants reporting an increase in alcohol use in Canada (33.4% vs. 22.9% for decrease) and a higher proportion reporting a decrease in France (33.5% vs. 21.4% for increase). Similar trends of changes in alcohol use among young adults were found in previous surveys in Canada [16,46,47] and France [48], mostly during the lockdown periods in spring and winter 2020. Previous researchers have attempted to explain the bidirectional changes in alcohol use patterns among young adults during the pandemic, with some hypothesizing that feelings of boredom, lack of routine, and loneliness were the main reasons for the increase, while others reported that self-care motives and lack of social gathering were associated with a decrease [5,7,49].

Our findings provide new insights into the socio-demographic characteristics of young adults who reported changes in their alcohol use due to the COVID-19 pandemic, including with regard to how these characteristics vary across study contexts. In both countries, young men and those with a high level of education had higher odds of reporting an increase. Conversely, racialized youth living in Canada had higher odds of reporting a decrease in their alcohol consumption. A similar result was observed in France where descendants of immigrants had lower odds of reporting an increase. These differences according to gender, education, and ethno-racial identity are consistent with previous COVID-19 studies conducted among young adults and students [28,47,50,51]. However, other socio-demographic characteristics are distributed differentially across countries. While no significant age differences were observed in France, adults aged 25 to 29 were more likely to report an increase in alcohol use in Canada compared to those aged 18 to 19. This may be related to the differences between Canada and France in terms of alcohol regulation laws and enforcement. In Canada, alcohol use is highly regulated (e.g., only available at licensed retailers, control of the legal drinking age, restrictions on consuming alcohol in public spaces), which makes access to alcohol more difficult for younger age groups compared to France where alcohol is available at any convenience store, can be consumed outdoors, and less restrictive proof-of-age controls are applied, especially in small pubs and shops that are easily accessible for youth [52,53]. In France, living away from family was associated with reporting an increase, a result that is consistent with previous surveys among students before [54,55] and during COVID-19 lockdown periods [21,51]. Young adults who live away from the family home are not influenced by parents’ norms and guidance, which may reduce access to and use of alcohol [56], and therefore may have more opportunities to interact with their peers, a social environment where alcohol is more commonly used. This association was not observed in Canada where participants who lived alone or with a partner were more likely to report no change. This contextual difference might be related to the socio-cultural differences in how young adults perceive and experience their transition from living with family to living more independently. At this juncture, further research is required to understand the broader contextual factors shaping this association.

Furthermore, our findings show that COVID-related stressors seem to have a protective effect on alcohol consumption among young adults. In both countries, participants who have been tested for COVID-19 were more likely to report a decrease in their alcohol use. While this result contrasts with findings showing that alcohol use was a risk factor for COVID-19 diagnosis among US students [15,25,57,58], this association may also reflect self-preventive behaviors of young adults who, as a result of their exposure to the virus and their experience with COVID-19 testing services, may be more likely to isolate themselves and limit their social interactions. In line with this hypothesis, young adults who were highly compliant with socially restrictive measures had higher odds of reporting a decrease in alcohol use. These findings also align with previous evidence, which showed a reduction in overall consumption of alcohol among youth during periods of COVID-19 restrictions in spring 2020 [18,26,59,60,61]. This may be explained by the fact that there were fewer opportunities for young adults to attend social gatherings or to drink socially with their peers. It is also important to note that these COVID-19 stressors were associated with an increase in alcohol use in Canada, suggesting that the experience of isolation may influence young adults’ alcohol patterns differently, including toward more consumption [17]. The association between COVID-19 stressors and changes in alcohol use in both directions could also have been influenced by the negative impact of the COVID-19 lockdown measures (e.g., closures of bars and nightclubs, temporary bans) on alcohol availability. However, many governments—including Canada and France—implemented minor restrictions on access to and affordability of alcohol in early 2020 [62,63] and relaxed regulations on alternative modes of supply, such as online sales and home delivery [64]. Furthermore, prior work reported a significant decline in the perceived availability of alcohol among US adolescents after the implementation of social distancing policies in 2020, but it also indicated that the prevalence of binge drinking did not change in their sample, suggesting a more nuanced picture regarding the relationship between alcohol availability and changes in alcohol use patterns among youth [65].

Other subsequent conditions that were not captured in our study may contribute to this adverse effect on alcohol use, such as social support, physical activity, and parental alcohol use. For instance, our data show that income loss influenced alcohol use in both directions in Canada, whereas no associations were observed in France. Previous COVID-19 studies with youth in North America documented that job insecurity, loss of employment, and financial concerns were associated with increased alcohol use [27,66]. In the opposite direction, participants who have lost income may have fewer financial resources to spend on alcohol or may choose to save money for other priority needs (e.g., rent, food) [5].

Our analysis also reveals that mental health and substance use factors were associated with increases in alcohol consumption in both countries, suggesting that young adults living in Canada and France experienced a similar context of stress and uncertainty due to the COVID-19 pandemic. We found a threshold effect when examining the association with depressive symptoms, such that only those with severe symptoms (in Canada) or moderately severe symptoms (in France) were more likely to report an increase in their alcohol use. This finding may be interpreted with bidirectional possibilities. Alcohol can be used as a coping strategy for depressive thoughts (i.e., self-medication theory) [67]. For example, a longitudinal study documented that young adults with more depressive symptoms before the pandemic were more resistant to reduce their frequency of alcohol use following the COVID-19 pandemic [68]. Conversely, higher alcohol consumption may also contribute to increases in risk of depressive symptoms [69], although this risk might be more likely to be experienced by those who had problematic use of alcohol before the pandemic (e.g., heavy drinking behaviors). In line with prior research [28], we also found that participants using tobacco or cannabis were more likely to report an increase in their alcohol use. These substances are closely interrelated, and co-use of two substances (e.g., alcohol–tobacco, alcohol–cannabis) has been tied to a greater risk of problematic alcohol use relative to single-alcohol use [70,71,72].

Our study also has some limitations. First, our study data were collected via convenience sampling and are not nationally representative of all young adult alcohol users in Canada or France. To account for this representation bias, we applied survey weights to adjust the sample distribution to the respective population distribution of each country according to age, gender, and province/region of residence. Second, we used self-reported measures of mental health (i.e., depressive symptoms) and substance use (i.e., tobacco, cannabis, and alcohol use), which are usually subject to underreporting and may be influenced by the social desirability bias. Nevertheless, Minhas and colleagues (2021) demonstrated that self-reported changes in alcohol use frequency corresponded to longitudinal changes in drinking among young adults in Canada [73]. Third, we did not collect the reasons for participants’ changes in alcohol use, which limits our interpretation of the key pandemic-related experiences that led to these changes. Lastly, the cross-sectional design of this study does not allow for testing causal effects.

Our study has implications for alcohol use interventions in the context of a global public health crisis. Our findings showed that changes in alcohol use of young adults varied according to socio-demographic characteristics (i.e., age, gender, ethno-racial identity, education), suggesting that the COVID-19 pandemic has affected youth differently according to their socio-demographic discrepancies and heterogeneous alcohol use experiences. For example, in our study, young men and older age groups were more likely to report increased alcohol use, while ethno-racial minority groups were more inclined to decrease their alcohol consumption during the first year of the pandemic. These results underscore the need to develop diverse alcohol use interventions that are targeted and tailored to the different social experiences and health needs of those specific sub-groups of young adults.

While our findings identified how the various social determinants of health (including living and socio-economic conditions, mental health, and substance use concerns) are important risk factors, it is also essential to account for the contextual factors related to public health crisis, which may affect the social behaviors of young adults (e.g., time in isolation, less social interaction), including their alcohol use patterns. Our findings also underscore the importance of investigating country-specific factors (e.g., regulation measures) in alcohol use patterns to best adapt and scale up alcohol use interventions for young adults. In light of the association between mental health and alcohol use, additional efforts are needed to strengthen the integrated approaches, two-way screening programs, and combined prevention campaigns.

## 5. Conclusions

More than half of young adults reported increases or decreases in alcohol use frequency during the first year of the COVID-19 pandemic. Efforts are needed to develop integrated alcohol-use prevention resources and support for young adults, which address both their mental health and substance use health-related needs and concerns. Further longitudinal research is warranted to understand how and why alcohol use patterns changed during and after the COVID-19 pandemic among young adults.

## Figures and Tables

**Table 2:** Weighted multivariate logistic regression analysis of factors associated with self-reporting a decrease and an increase in alcohol use because of the COVID-19 pandemic among young adults in Canada (*n*= 2725) and France (*n* = 2328).

		Canada				France			
		Reference Group: No Change				Reference Group: No Change			
		Decrease		Increase		Decrease		Increase	
		AOR (95% CI)	*p*-Value	AOR (95% CI)	*p*-Value	AOR (95% CI)	*p*-Value	AOR (95% CI)	*p*-Value
***Socio-demographic*** ***characteristics***									
**Age (years)**									
	18–19	Ref.		Ref.		Ref.		Ref.	
	20–24	0.95 (0.73, 1.25)	0.7	1.19 (0.91, 1.55)	0.2	0.96 (0.74, 1.23)	0.7	1.14 (0.81, 1.60)	0.5
	25–29	0.93 (0.68, 1.29)	0.7	**1.61 (1.19, 2.19)**	**0.002**	0.75 (0.54, 1.05)	0.1	1.33 (0.86, 2.05)	0.2
**Gender identity**									
	Woman	Ref.		Ref.		Ref.		Ref.	
	Man	1.08 (0.89, 1.32)	0.4	**1.52 (1.28, 1.82)**	**<0.001**	**1.28 (1.08, 1.51)**	**0.004**	**1.45 (1.18, 1.78)**	**<0.001**
	Non-binary/other gender identity ^$^	1.03 (0.74, 1.44)	0.8	0.93 (0.68, 1.27)	0.6	0.82 (0.54, 1.24)	0.3	1.2 (0.76, 1.90)	0.4
**Sexual orientation**									
	Straight/heterosexual	Ref.		Ref.		Ref.		Ref.	
	Bisexual	0.93 (0.75, 1.15)	0.5	0.85 (0.69, 1.04)	0.12	1.23 (0.99, 1.53)	0.067	1.31 (1.00, 1.71)	0.051
	Other sexual minority ^£^	0.97 (0.75, 1.25)	0.8	**0.78 (0.62, 0.99)**	**0.041**	0.81 (0.65, 1.01)	0.064	0.84 (0.64, 1.11)	0.2
**Province of residence** **(only Canada)** ^¶^									
	Ontario	Ref.		Ref.					
	Atlantic	1.00 (0.74, 1.36)	>0.9	0.98 (0.73, 1.30)	0.9				
	British Columbia	1.09 (0.85, 1.40)	0.5	1.13 (0.89, 1.43)	0.3				
	Prairies	0.79 (0.61, 1.02)	0.072	1.17 (0.93, 1.48)	0.2				
	Quebec	**1.48 (1.15, 1.90)**	**0.002**	**1.41 (1.12, 1.79)**	**0.004**				
	Territories	1.15 (0.58, 2.27)	0.7	0.66 (0.37, 1.18)	0.2				
**Region of residence** **(only France)** *^ ^^*									
	Ile-de-France					Ref.		Ref.	
	Nord Est					0.89 (0.69, 1.15)	0.4	1.14 (0.84, 1.55)	0.4
	Ouest					0.81 (0.63, 1.04)	0.1	**0.64 (0.46, 0.89)**	**0.008**
	Sud Est					**0.77 (0.61, 0.97)**	**0.029**	1.01 (0.76, 1.35)	>0.9
	Sud Ouest					0.83 (0.65, 1.05)	0.12	0.82 (0.60, 1.11)	0.2
**Area of living**									
	Large urban center	Ref.		Ref.		Ref.		Ref.	
	Medium city or town	0.91 (0.72, 1.15)	0.4	0.9 (0.73, 1.11)	0.3	0.87 (0.71, 1.06)	0.2	**1.32 (1.03, 1.70)**	**0.031**
	Small city or rural area	0.93 (0.73, 1.19)	0.6	0.89 (0.71, 1.12)	0.3	**0.81 (0.66, 1.00)**	**0.055**	1.12 (0.87, 1.44)	0.4
**Ethno-racial identity** **(only Canada)** *^§^*									
	Non-racialized	Ref.		Ref.					
	Indigenous	0.66 (0.39, 1.12)	0.12	1.2 (0.83, 1.76)	0.3				
	Racialized, non-Indigenous	**1.44 (1.04, 1.99)**	**0.027**	**0.65 (0.46, 0.91)**	**0.012**				
**Descendants of immigrants (only France)**									
	No					Ref.		Ref.	
	Yes					0.83 (0.65, 1.08)	0.2	**0.68 (0.50, 0.93)**	**0.015**
**Foreign-born status**									
	No	Ref.		Ref.		Ref.		Ref.	
	Yes	**0.66 (0.49, 0.89)**	**0.006**	0.94 (0.70, 1.26)	0.7	1.29 (0.88, 1.90)	0.2	0.92 (0.59, 1.43)	0.7
**Education**									
	High school or college	Ref.		Ref.		Ref.		Ref.	
	University (some, completed, or above)	**1.58 (1.27, 1.96)**	**<0.001**	1.19 (0.99, 1.45)	0.067	**1.47 (1.19, 1.82)**	**<0.001**	**1.48 (1.14, 1.91)**	**0.003**
**Employment status**									
	Employed	Ref.		Ref.		Ref.		Ref.	
	Student (incl. employed)	0.93 (0.75, 1.17)	0.6	1.04 (0.85, 1.28)	0.7	1.13 (0.90, 1.41)	0.3	0.94 (0.72, 1.23)	0.7
	Unemployed	0.73 (0.52, 1.04)	0.078	**0.74 (0.55, 1.00)**	**0.049**	0.95 (0.71, 1.28)	0.8	1.23 (0.89, 1.70)	0.2
**Living arrangements**									
	With family members	Ref.		Ref.		Ref.		Ref.	
	Alone	**0.64 (0.47, 0.87)**	**0.004**	0.83 (0.63, 1.09)	0.2	1.15 (0.93, 1.44)	0.2	**1.67 (1.25, 2.23)**	**<0.001**
	With partner/spouse	**0.73 (0.57, 0.94)**	**0.014**	1.12 (0.88, 1.41)	0.4	0.93 (0.72, 1.19)	0.5	**1.56 (1.16, 2.10)**	**0.004**
	With friends/ roommates	1.24 (0.98, 1.58)	0.072	1.14 (0.91, 1.42)	0.3	1.26 (0.97, 1.63)	0.08	**2.00 (1.45, 2.75)**	**<0.001**
***COVID-19-related stressors***									
**Experience of COVID-19 testing**									
	No	Ref.		Ref.		Ref.		Ref.	
	Yes	**1.33 (1.10, 1.59)**	**0.002**	**1.20 (1.01, 1.43)**	**0.036**	**1.36 (1.16, 1.59)**	**<0.001**	1.09 (0.89, 1.32)	0.4
**Level of compliance with COVID-19 social** **restrictions measures**									
	Low	Ref.		Ref.		Ref.		Ref.	
	Medium	**1.76 (1.36, 2.28)**	**<0.001**	1.17 (0.94, 1.46)	0.2	1.19 (0.99, 1.41)	0.057	1.15 (0.93, 1.43)	0.2
	High	**2.15 (1.64, 2.82)**	**<0.001**	**1.39 (1.10, 1.75)**	**0.006**	**2.17 (1.71, 2.76)**	**<0.001**	1.03 (0.74, 1.42)	0.9
**Income loss due to the COVID-19 pandemic**									
	No	Ref.		Ref.		Ref.		Ref.	
	Yes	**1.34 (1.12, 1.60)**	**0.001**	**1.42 (1.21, 1.68)**	**<0.001**	1.08 (0.90, 1.30)	0.4	1.15 (0.93, 1.41)	0.2
***Mental health and substance use factors***									
**Depressive symptoms**									
	Mild	Ref.		Ref.		Ref.		Ref.	
	Minimal	**0.52 (0.38, 0.72)**	**<0.001**	**0.51 (0.38, 0.68)**	**<0.001**	0.81 (0.66, 1.00)	0.053	**0.43 (0.31, 0.58)**	**<0.001**
	Moderate	1.03 (0.80, 1.31)	0.8	1.02 (0.81, 1.29)	0.9	**0.78 (0.63, 0.97)**	**0.023**	0.87 (0.67, 1.13)	0.3
	Moderate–severe	1.01 (0.77, 1.32)	>0.9	1.14 (0.88, 1.47)	0.3	0.99 (0.78, 1.27)	>0.9	**1.37 (1.02, 1.85)**	**0.038**
	Severe	1.13 (0.86, 1.49)	0.4	**1.52 (1.17, 1.97)**	**0.001**	1.24 (0.92, 1.67)	0.2	**1.96 (1.41, 2.73)**	**<0.001**
**Tobacco use**									
	Non-use	Ref.		Ref.		Ref.		Ref.	
	Occasional smoking	1.20 (0.91, 1.58)	0.2	**1.43 (1.12, 1.82)**	**0.004**	**1.65 (1.33, 2.03)**	**<0.001**	**1.52 (1.13, 2.04)**	**0.005**
	Regular smoking	1.41 (0.89, 2.24)	0.15	**2.82 (1.96, 4.05)**	**<0.001**	1.14 (0.92, 1.40)	0.2	**2.13 (1.65, 2.76)**	**<0.001**
**Cannabis use**									
	No	Ref.		Ref.		Ref.		Ref.	
	Yes	1.05 (0.87, 1.26)	0.6	**2.00 (1.67, 2.38)**	**<0.001**	1.10 (0.92, 1.32)	0.3	**1.83 (1.45, 2.32)**	**<0.001**

Notes: AOR: Adjusted Odds Ratio, CI: Confidence Interval, Ref.: Reference group. Analyses were adjusted for all characteristics listed in the table. Bold indicates significant *p* values at ≤0.05. ^$^ Other gender identity included intersex, two-spirit (only for Canada), and other gender identity with an open-text box. ^£^ Other sexual minority included asexual, pansexual, queer, two-spirit (only for Canada), and other sexual identity with an open-text box. ^§^ Participants who selected any ethno-racial identity (one or more) other than white or Indigenous were classified as racialized, non-Indigenous. The category “non-racialized” includes young adults who selected “white” only and those who reported “white and Latino” or “white and Middle-Eastern” as per the definition in the Canadian Employment Equity Act. The Indigenous category includes those who self-identify as First Nations, Métis, Inuk/Inuit descents. ^¶^ Atlantic: New Brunswick, Newfoundland and Labrador, Prince Edward Island, Nova Scotia; Prairies: Alberta, Manitoba, Saskatchewan; Territories: Nunavut, Yukon, Northwest Territories. ^^^ Nord Est: Grand-Est, Hauts-de-France, Bourgogne Franche-Comté; Sud Est: Auvergne-Rhône-Alpes, Provence-Alpes-Côte-d’Azur, Corse; Sud Ouest: Nouvelle Aquitaine, Occitanie; Ouest: Bretagne, Centre Val-de-Loire, Pays de la Loire, Normandie. Participants from overseas departments were included in the sub-category Sud Est.

## Data Availability

Fully anonymized data are available on request from the FOCUS study principal investigators (Rod Knight at rod.knight@bccsu.ubc.ca and Marie Jauffret-Roustide at marie.jauffret-roustide@inserm.fr).
